# Optimum solution of power flow problem based on search and rescue algorithm

**DOI:** 10.1038/s41598-024-78086-y

**Published:** 2024-11-17

**Authors:** Essam H. Houssein, Alaa A. K. Ismaeel, Mokhtar Said

**Affiliations:** 1 Faculty of Computers and Information, Minia University, Minia, 61519 Egypt; 2 Faculty of Computer Studies (FCS), Arab Open University (AOU), 130, Muscat, Oman; 3 Faculty of Science, Minia University, Minia, 61519 Egypt; 4 Electrical Engineering Department, Faculty of Engineering, Fayoum University, Fayoum, 43518 Egypt

**Keywords:** Search and rescue algorithm, Optimal power flow, Power system, Computer science, Energy science and technology

## Abstract

In order to solve the optimal power flow (OPF) problem, a unique algorithm based on a search and rescue method is applied in this study. For the OPF problem under three objective functions, the SAR offers a straightforward and reliable solution. The three objective functions are used to minimize the fuel cost, power loss and voltage deviation as a single objective function. The OPF problem for benchmark test system, including the IEEE-14 bus, IEEE-30 bus, and IEEE-57 bus, are solved by the Search and Rescue algorithm (SAR) under specific objective functions that are determined by the operational and economic performance indices of the power system. To demonstrate the efficacy and possibilities of the SAR algorithm, SAR is contrasted with alternative optimization techniques such as harmony search algorithm, gradient method, adaptive genetic algorithm, biogeography-based optimization, Artificial bee colony, gravitational search algorithm, particle swarm optimization, Jaya algorithm, enhanced genetic algorithm, modified shuffle frog leaping algorithm, practical swarm optimizer, Moth flam optimizer, whale and moth flam optimizer, grey wolf optimizer, cheap optimization algorithm and differential evolution algorithm. The value of minimum power losses based on SAR technique is equal to 0.459733441487247 MW for IEEE-14 bus. The value of minimum total fuel cost based on SAR technique is equal to 8051.12225602148 $/h for IEEE-14 bus. The value of minimum voltage deviation based on SAR technique is equal to 0.0357680148269292 for IEEE-14 bus. The value of minimum power losses based on SAR technique is equal to 2.71286428848434 MW for IEEE-30 bus. The value of minimum total fuel cost based on SAR technique is equal to 798.197578585806 $/h for IEEE-30 bus. The value of minimum voltage deviation based on SAR technique is equal to 0.0978069572088536 for IEEE-30 bus. The value of minimum total fuel cost based on SAR technique is equal to 38017.7691758245 $/h for IEEE-57 bus. The acquired results for the OPF compared to all competitor algorithms in every case of fitness function demonstrate the superiority of the SAR method.

## Introduction

Technical economical operation is the most important issue in electric utilities. The scheduling of the energy generation to reduce the cost of energy generation with reliability of meet al.l operating load demand is the main objective of power system operation. The optimal operation in power system is a non-linear system and a complex problem in optimization, that is solved using optimal power flow as a main objective function. The optimal power flow (OPF) is solved in two ways, meta-heuristic optimization algorithms and mathematical methods. Some variables in the power system need control by preserving its value in the constraints of system to achieve the best schedule in optimal power flow problem^1–5^.

The mathematical methods used in OPF solution are several such as linear and non-linear programming (LP-NLP)^6,7^, interior point method^8^, quadratic programming^9^, Newton-based method^10^ and gradient projection method^2^. These methods have demerits such as accuracy is not guaranteed, convergence is poor, complexity of methods is high and initial guess is required for some method. Therefore, the dependence on metaheuristic algorithms are necessary to overcome these demerits.

Several metaheuristic algorithms are used in OPF such as sine cosine algorithm^5^, genetic algorithm and its improvement^11–13^, practical swarm optimization and its improvement^14–16^, hybrid algorithm of the shuffle frog leaping algorithm and practical swarm optimization algorithm^17^, shuffle frog leaping algorithm^18^, biogeography based optimizer^19^, a chaotic invasive weed optimization algorithm^20^, teaching-learning-based optimization^21^, modified imperialist competitive algorithm^22^, moth swarm algorithm^23^, a modified bacteria foraging algorithm^24^, the backtracking search algorithm^25^, Social spider optimization^26^, improved adaptive differential evolution^27^, decentralized consensus algorithm^28^, hybrid particle swarm and slap optimization algorithm^29^, an improved adaptive differential evolution^27^, an Analytical adaptive distributed multi-objective optimization algorithm^30^, A heuristic benders-decomposition-based algorithm^31^ and an adaptive multiple teams perturbation-guiding Jaya algorithm^32^.

A number of effective metaheuristic algorithms have been put out recently to effectively address a range of optimization issues^33–37^. The African Vultures Optimization Algorithm (AVOA) was introduced in^33^ and is used to solve engineering problems and benchmark functions. AOA^34^ is an algorithm for arithmetic optimization that was developed to tackle a variety of mechanical issues, including tension/compression spring design and welding beam design. The artificial gorilla troops’ optimizer (GTO) was introduced in^35^ and is used to resolve engineering challenges and benchmark routines. AHA, or the artificial hummingbird algorithm, was developed^36^ to tackle difficult engineering design problems and numerical test functions. The marine predator’s algorithm (MPA) was presented in^37^ as a solution to engineering benchmarks, test functions, and practical engineering design issues. The majority of these, nevertheless, have never been looked into in MaOPF issues. Numerous optimization issues across multiple domains, including Optimal power flow^38–41^, identification of solar cell parameters^42–45^, economic load dispatch^46–49^ and several issues. In this work, the search and rescue optimization will be used for solving the problem of optimal power flow. IEEE 57-bus, IEEE 30-bus and IEEE 14-bus systems are used for testing the proposed optimization algorithm. Comparison between the simulation results and the reported results from literature works is performed for measure the effectiveness of the proposed algorithm.

The main contributions of this work can be summarized as follow:

- The problem of OPF will be solved with a new optimization algorithm called search and rescue algorithm (SAR).
- Three single fitness functions and multi-objective function are applied on IEEE 57-bus, IEEE 30-bus and IEEE 14-bus system as a cases study.
- The first objective function is minimizing the total cost of fuels associated with power production.
- The second objective function is minimizing the voltage deviation.
- The third objective function is minimizing the total power losses.
- The fourth objective function is minimizing the cost in addition to the voltage deviation as a multi-objective function.
- The effectiveness and superiority of the proposed algorithm (SAR) in solving OPF problem is performed by comparison its results with most recently previous optimization algorithms such as harmony search algorithm, gradient method, adaptive genetic algorithm, biogeography-based optimization, Artificial bee colony, gravitational search algorithm, particle swarm optimization, Jaya algorithm, enhanced genetic algorithm, modified shuffle frog leaping algorithm, practical swarm optimizer, Moth flam optimizer, whale and moth flam optimizer, grey wolf optimizer, cheap optimization algorithm and differential evolution algorithm.

This work is structured as follows: section two describes the optimal power flow problem. In Section Three, the suggested SAR algorithm is described. The cases study results are analyzed in part four, and the conclusion is presented in section five.

## Analysis of the OPF problem

The OPF problem is conceptualized as a kind of nonlinear optimization problem. Finding the optimal configurations for the power system control variables that maximize predetermined system objectives while observing system restrictions is its main goal^5,58^. The issue is written mathematically as^5,58^:

1 $$Min \ F(X,U)$$

2 $$g(X,U)= 0$$

3 $$h(X,U) \leq 0$$

where, respectively, U and X are the vectors of the control and dependent variables. In Eq. (1), the fitness function to be optimized is denoted by$\:\:F\left(X,U\right)$. The equality and inequality system constraints are represented by the functions $\:g\left(X,\:U\right)$ and $\:h\left(X,\:U\right)$ respectively.

The balance of active and reactive powers at all system buses is a requirement for equality. The inequality constraints entail maintaining the load bus voltage ($\:{V}_{Li}$), switchable VAR compensations ($\:{Q}_{c}$), transformer tap (T) and generator voltage ($\:{V}_{G}$) limits.

The following system variables make up the state vector X^5,58^:

1. The slack bus generator’s actual output power ($\:{P}_{G1}$).
2. The load-bus voltage level ($\:{V}_{L}$).
3. The generator produces $\:{Q}_{G}$ of reactive power.
4. The transmission lines’ power flow ($\:{S}_{NTL}$)

The state vector X can be expressed mathematically as:

4 $$\:{X}^{T}=\left[{P}_{G1},\:{V}_{LL1},\dots\:,{V}_{LLN},.{Q}_{G1},\dots\:{Q}_{GN},{S}_{1TL},\dots\:{S}_{NTL}\right]$$

where NG, NL, NTL, and STL stand for respective numbers of generators, load buses, transmission lines, transmission line loading.

The vector of decision variables (U) is involving of generator voltages $\:{V}_{G\:}$, real power outputs from generator $\:{P}_{G\:}$excluding the slack, Shunt VAR compensations $\:{Q}_{c\:}$and tap settings $\:T\:$. As a result, the control parameters vector (U) can be described as the following form^5,58^:

5 $$\:{U}^{T}=\left[{P}_{G2}\dots\:{P}_{GN}\:,\:{V}_{G1}\dots\:{V}_{GN},.{T}_{1}\dots\:{T}_{NT},{Q}_{C1}\dots\:{Q}_{CNC}\right]$$

where NT and NC stand for the relative numbers of regulating transformers and shunt VAR compensators.

### OPF fitness function

In order to solve the OPF problem, four objectives are tested in this work: an economic problem (i.e., reducing the total fuel costs associated with power production); an operational issue regarding the voltage deviation; a practical challenge regarding the power losses; and a practical challenge regarding the cost in addition to the voltage deviation as a multi-objective function.

#### The first OPF fitness function (OPFFF1)

The OPFFF1 is to reduce the overall fuel costs for the contracted power generators. It can be stated as follows^5,58^:

6 $$\:{K}_{f}=\sum\:_{i=1}^{NG}\left({a}_{i}+{b}_{i}{P}_{i}+{c}_{i}{P}_{i}^{2}\right)\:\:\:\:\:\:\:\:\:\:\:\:\:\:$$

where $\:{a}_{i}$, $\:{c}_{i}\:$, and $\:{b}_{i}$ are the $\:{i}^{th}$ generator’s cost coefficients. where $\:{c}_{im}$,$\:\:{b}_{im}$ and $\:{a}_{im}\:$ are the coefficients of $\:{i}^{th}$ generator cost according to fuel type m.

#### The second OPF fitness function (OPFFF2)

The OPFFF2 is to reduce the active power transmission losses. It can be stated as follows^5,58^:

7 $$\:{P}_{loss}=\sum\:_{i,j\in\:{N}_{b}}{g}_{ij}\left({V}_{i}^{2}+{V}_{j}^{2}-2{V}_{i}{V}_{j}\text{cos}{\theta\:}_{ij}\right)$$

where $\:{\theta\:}_{ij}$ is the voltage angle difference between buses $\:i\:and\:j\:\:$, $\:{g}_{ij}$ is the conductance of the branch between buses $\:i\:and\:j\:$, The magnitudes of voltage at buses $\:i\:and\:j$ are $\:{V}_{i}\:and\:{V}_{j}\:$ respectively.

#### The third OPF fitness function (OPFFF3)

The OPFFF3 is to reduce the voltage deviation of each load bus. The voltage deviation is formally represented as follows^5,58^:

8 $$\:{K}_{V}=\sum\:_{i=1}^{NG}\left(\left|{V}_{Li}-{V}_{ref}\right|\right)\:\:\:\:\:\:\:\:\:\:\:\:\:\:\:\:\:$$

#### The fourth OPF fitness function (OPFFF4)

The OPFFF4 is a multi-objective fitness function. It aims to reduce the fuel cost and the voltage deviation. The OPFFF4 is formally represented as follows^5,58^:

9 $$\:{K}_{PV}=\sum\:_{i=1}^{NG}\left({a}_{i}+{b}_{i}{P}_{i}+{c}_{i}{P}_{i}^{2}\right)+We\sum\:_{i=1}^{NG}\left(\left|{V}_{Li}-{V}_{ref}\right|\right)\:\:\:\:\:\:\:$$

where the weighting factor is represented by $\:We$ = 200^58^. Equation (9) integrates two weighted objectives into a single equation to efficiently handle the multi-objective problem. This objective function seeks to simultaneously minimize the fuel cost and the voltage variation.

### Power system operational constraints

For varied operating scenarios, equality and inequality constraints, two forms of operational constraints for power systems, must be taken into account:

#### Equality constraints

The active and reactive power balance is represented by the power balance constraints. These restrictions are represented as follows^5,58^:

10 $$\:{P}_{Gi}-{P}_{Li}-{P}_{Loss}=0$$

11 $$\:{Q}_{Gi}-{Q}_{Li}-{Q}_{Ci}-{Q}_{Loss}=0\:\:\:\:\:\:\:\:\:\:$$

Where, $\:{P}_{Gi}\:$, $\:{Q}_{Gi}\:$: The $\:ith$ generator bus’s active and reactive power, respectively $\:{P}_{Li}\:$, $\:{Q}_{Li}\:$: The $\:ith$ load bus’s active and reactive power, respectively. Total active and reactive network losses are denoted by $\:{P}_{Loss}\:$ and $\:{Q}_{Loss}\:$, respectively. $\:{Q}_{Ci}$ is the reactive compensation power for bus $\:i$.

#### Inequality constraints

The constraints of voltages of generation buses are as follow^5,58^:

12 $$\:{V}_{Gi}^{min}\le\:{V}_{Gi}\le\:{V}_{Gi}^{max}\:\:\:\:\:\:\:\:\:\:\:\:\:\:\:\:\:i=1,\dots\:\dots\:NG\:$$

The constraints of reactive generation power from buses are as follow:

13 $$\:{Q}_{Gi}^{min}\le\:{Q}_{Gi}\le\:{Q}_{Gi}^{max}\:\:\:\:\:\:\:\:\:\:\:\:\:\:\:\:\:i=1,\dots\:\dots\:NG\:\:\:\:\:\:\:\:$$

The constraints of transformer taps are as follow:

14 $$\:{T}_{k}^{min}\le\:{T}_{k}\le\:{T}_{k}^{max}\:\:\:\:\:\:\:\:\:\:\:\:\:\:\:\:\:k=1,\dots\:\dots\:NT$$

The constraints of shunt compensation units are as follow:

15 $$\:{Q}_{cm}^{min}\le\:{Q}_{cm}\le\:{Q}_{cm}^{max}\:\:\:\:\:\:\:\:\:\:\:\:\:\:\:\:\:m=1,\dots\:\dots\:NC\:\:\:$$

The constraints of load buses voltage are as follow:

16 $$\:{V}_{Lj}^{min}\le\:{V}_{Lj}\le\:{V}_{Lj}^{max}\:\:\:\:\:\:\:\:\:\:\:\:\:\:\:\:j=1,\dots\:\dots\:NG\:$$

## Search and Rescue Optimization Method

This section presents the mathematical model of SAR algorithm to solve the ‘‘minimization problem”. In which, the humans’ position confronts the solution for the optimization problem whereas the clue significance reached in this position denotes the fitness for that solution. An optimal solution indicates a clue with high significance and vice versa^50^.

For solving optimization issues, Shabani, Amir, et al.^50^ have proposed a new metaheuristic algorithm called Search and rescue optimization technique (SAR). In SAR, the fitness for the solution is obtained from the human position that corresponds to the solution of the significance of the clue found. A better solution represents a more significant clue, and vice versa. An overview of the main steps for the SAR algorithm is summarized as follows:

### Clues

Information on clues collected by group members during the search operation. Some clues are left, but the associate information is stored in a memory matrix called “matrix M” to utilize to select the most significant clues. Whereas the positions of humans are saved in a matrix called “matrix X”, with $\:N\times\:D$ is the problem’s dimension and the number of group members denotes by N. But the found clues are generated using Eq. (17) to act on the Clues matrix called “matrix C”. Moreover, in each human search phase, the previous three matrices (X, M, and C) are updated.

17 $$\:C=\left[\begin{array}{c}X\\\:M\end{array}\right]=\left[\begin{array}{ccc}{X}_{11}&\cdots&{X}_{1Z}\\ \vdots& \ddots & \vdots\\ {X}_{Y1}&\cdots&{X}_{YZ}\\ {M}_{11}& \cdots & {M}_{1Z}\\\vdots &\:\ddots\:&\vdots \\\:{M}_{Y1}&\:\cdots & {M}_{YZ}\end{array}\right]\:$$

where, memory and humans’ positions matrices are denoted by M and X, respectively. The position of the 1st dimension is denoted by $\:{X}_{N1}$ for the $\:{N}^{th}$ human and the position of the $\:{D}^{th}$ dimension is denoted by $\:{M}_{1D}\:$for the1st memory.

### Social phase

The clue among the clues found is randomly calculated using Eq. (18) to obtain the search direction.

18 $$\:{SZ}_{i}=\left({X}_{i}-{C}_{k}\right),\:k\ne\:i\:$$

where the position of the $\:{i}^{th}$ human is indicated by $\:{X}_{i}$, the position of the clue $\:{k}^{th}$ is indicated by $\:{C}_{k}$, and the search direction for the $\:{i}^{th}$ human is indicated by $\:{SD}_{i}$ is a random integer number within [1, 2 N] and$\:\:k\ne\:i$.

The binomial crossover operator is applied to ensure that all dimensions of $\:{X}_{i}$ are not changed. Equation (19) is used for the social phase to compute the new position of the $\:{i}^{th}$ human in all dimensions as follows:

19 $${\acute{X}}_{ij}=\left\{\begin{array}{cc}\left\{\begin{array}{c}{C}_{kj}+r1\times\:\left({X}_{ij}-{C}_{kj}\right)\:if\:f\left({C}_{k}\right)>f\left({X}_{i}\right)\\\:{X}_{ij}+r1\times\:\left({X}_{ij}-{C}_{kj}\right)\:\:\:\:\:\:\:\:\:\:\:\:\:otherwise\end{array}\right.&\:if\:r2<SE\:or\:j={j}_{rand},\:j=1,\dots\:,Z\\\:{X}_{ij}\:\:\:\:\:\:\:\:\:\:\:\:\:\:\:\:\:\:\:\:\:\:\:\:\:\:\:\:\:\:\:\:\:\:\:\:&\:otherwise\end{array}\right.$$

where the $\:{j}^{th}$ dimension is the new position is denoted by $\:{\acute{X}}_{ij}$. The position of the $\:{j}^{th}$ dimension is denoted by $\:{C}_{kj}$for the clue $\:{k}^{th}$. The values of the objective function are defined by $\:f\left({C}_{k}\right)$) and $\:f\left({X}_{i}\right)$. $\:r1\:$is a uniformly distributed random number within [-1, 1] and is fixed for all dimensions. But $\:r2$ is a random number with a uniform distribution within [0, 1] and not fixed as $\:r1\:$A random integer number within [1 and D] is denoted by $\:{j}_{rand}$ and $\:SE$ is a parameter within [0, 1].

### Individual phase

The new position of the $\:{i}^{th}$ human search around their current position is computed as follows:

20 $${\acute{X}}_{i}={X}_{i}+r3\times\:\left({C}_{k}-{C}_{m}\right),i\ne\:k\ne\:m$$

where m and k are random numbers within [1, 2 N], and is set as $\:\text{i}\ne\:\text{k}\ne\:\text{m}$ to prevent movement along other clues. In addition, $\:r3$ is a uniformly distributed random number within [0, 1].

### Boundary control

The new position of the $\:{i}^{th}$ human, the social, and individual phases are modified using Eq. (21).

21 $$\:{\acute{X}}_{ij}=\left\{\begin{array}{cc}\left({X}_{ij}+{X}_{j}^{max}\right)/2&\:if\:{\acute{X}}_{ij}>{X}_{j}^{max}\\\:\left({X}_{ij}+{X}_{j}^{min}\right)/2&\:if\:{\acute{X}}_{ij}>{X}_{j}^{min}\end{array},\:j=1,\dots\:,Z\right.$$

where the values of the minimum and maximum threshold are denoted by $\:{X}_{j}^{min}$and $\:{X}_{j}^{max}$for the $\:{j}^{th}$ dimension.

### Updating information and positions

The new position and the random position of the memory matrix (M) are computed using Eq. (22) and Eq. (23) respectively.

22 $$\:{M}_{n}=\left\{\begin{array}{cc}{X}_{i}&\:if\:f\left({\acute{X}}_{i}\right)>f\left({X}_{i}\right)\\\:{M}_{n}&\:otherwise\end{array}\right.\:$$

23 $$\:{X}_{i}=\left\{\begin{array}{cc}{\acute{X}}_{i}&\:if\:f\left({\acute{X}}_{i}\right)>f\left({X}_{i}\right) \\\:{X}_{i}&\:otherwise\end{array}\right.\:$$

where $\:{M}_{n}\:$is the position of the $\:{n}^{th}$ stored clue in the memory matrix.

### Abandoning clues

For each group member, Unsuccessful Search Number (USN) is set to 0 for a human finds more significant clues else increased by 1 using Eq. (24).

24 $$\:{USN}_{i}=\left\{\begin{array}{cc}{USN}_{i}+1&\:if\:f\left({\acute{X}}_{i}\right)>f\left({X}_{i}\right)\\\:0\:\:\:\:\:\:\:\:\:\:\:\:\:\:\:&\:otherwise\end{array}\right.\:$$

where the number of times the $\:{i}^{th}$ human is denoted by $\:{USN}_{i}\:$. Equation (25) is used to change current solution with a random solution in the search space if $\:USN\:>\:MU$.

25 $$\:{X}_{ij}={X}_{j}^{min}+{r}_{4}\times\:\left({X}_{j}^{max}-{X}_{j}^{min}\right),j=1,\dots\:,Z\:$$

where $\:{\text{r}}_{4}$ is a uniformly distributed random number within [0, 1] and various with each dimension.

### The technique of constraint-handling

The penalty functions methods called $\:{\upepsilon\:}$ -constrained method is applied in SAR algorithm for a maximization problem based on Eq. (26).

26 $$\:\begin{array}{c}{X}_{1}\:is\:better\:than\:{X}_{2}:\:\:\:\:\:\:\:\:\:\:\:\:\:\:\:\:\:\:\:\:\:\:\:\:\:\:\:\:\:\:\:\:\:\:\:\:\:\:\:\:\:\:\:\:\:\:\:\:\:\\\:\left\{\begin{array}{cc}f\left({X}_{1}\right)>f\left({X}_{2}\right)&\:if\:G\left({X}_{1}\right)\le\:\epsilon\:\:and\:G\left({X}_{2}\right)\le\:\epsilon\:\\\:f\left({X}_{1}\right)>f\left({X}_{2}\right)&\:if\:G\left({X}_{1}\right)=G\left({X}_{2}\right)\\\:G\left({X}_{1}\right)>G\left({X}_{2}\right)&\:otherwise\end{array}\right.\end{array}\:$$

To controls the size of feasible space, the $\:{\upepsilon\:}$ parameter is used. It is calculated by Eq. (27).

27 $$\:\epsilon\:\left(t\right)=\left\{\begin{array}{cc}{G}_{\theta\:}{\left(1-\frac{t}{{T}_{c}}\right)}^{cp}&\:if\:t\le\:{T}_{c}\\\:0&\:otherwise\end{array}\right.\:$$

where $\:\text{t}$ is the number of the current iteration. The $\:{h}^{th}$ smallest violation of constraints denoted by$\:\:{G}_{\theta\:}$ in the initial population. $\:{T}_{c}\:$and $\:cp$ are two parameters used to truncate $\:\epsilon\:$ and control the speed of reducing feasible space, respectively.

In addition to constraint optimization problems, therefore, Eqs. (21), (22), (23), and (24) are modeled as follows:

28 $$\:\begin{array}{c}{\acute{X}}_{ij}=\:\:\:\:\:\:\:\:\:\:\:\:\:\:\:\:\:\:\:\:\:\:\:\:\:\:\:\:\:\:\:\:\:\:\:\:\:\:\:\:\:\:\:\:\:\:\:\:\:\:\:\:\:\:\:\:\:\:\:\:\:\:\:\:\:\:\:\:\:\:\:\:\:\:\:\:\:\:\:\:\\\:\left\{\begin{array}{cc}\left\{\begin{array}{c}{C}_{kj}+r1\times\:\left({X}_{ij}-{C}_{kj}\right)\:\:if\:{C}_{k}\:is\:better\:than\:{X}_{i}\\\:{X}_{ij}+r1\times\:\left({X}_{ij}-{C}_{kj}\right)\:\:\:\:\:\:\:\:\:\:\:\:\:\:\:\:\:\:\:\:\:\:\:\:\:\:otherwise\end{array}\right.&\:\begin{array}{c}if\:r2<SE\:or\:j=\:\\\:{j}_{rand},\:j=1,\dots\:,Z\end{array}\\\:{X}_{ij}\:\:\:\:\:\:\:\:\:\:\:\:\:\:\:\:\:\:\:\:\:\:\:\:\:\:\:\:\:\:\:\:\:\:\:\:\:\:\:\:\:\:\:\:\:\:\:\:\:\:\:\:\:&\:otherwise\end{array}\right.\end{array}$$

29 $$\:{M}_{n}=\left\{\begin{array}{cc}{\acute{X}}_{i}&\:if\:{\acute{X}}_{i}\:is\:better\:than\:{X}_{i}\\\:{M}_{n}&\:otherwise\end{array}\right.\:$$

30 $$\:{X}_{i}=\left\{\begin{array}{cc}{\acute{X}}_{i}&\:if\:{\acute{X}}_{i}\:is\:better\:than\:{X}_{i}\\\:{X}_{i}&\:otherwise\end{array}\right.$$

31 $$\:{USN}_{i}=\left\{\begin{array}{cc}0\:\:\:\:\:\:\:\:\:\:\:\:\:\:\:\:\:&\:if\:f\left({\acute{X}}_{i}\right)>f\left({X}_{i}\right)\\\:{USN}_{i}+1\:\:\:\:\:\:\:\:\:\:\:&\:otherwise\end{array}\right.\:$$

**Fig. 1:**
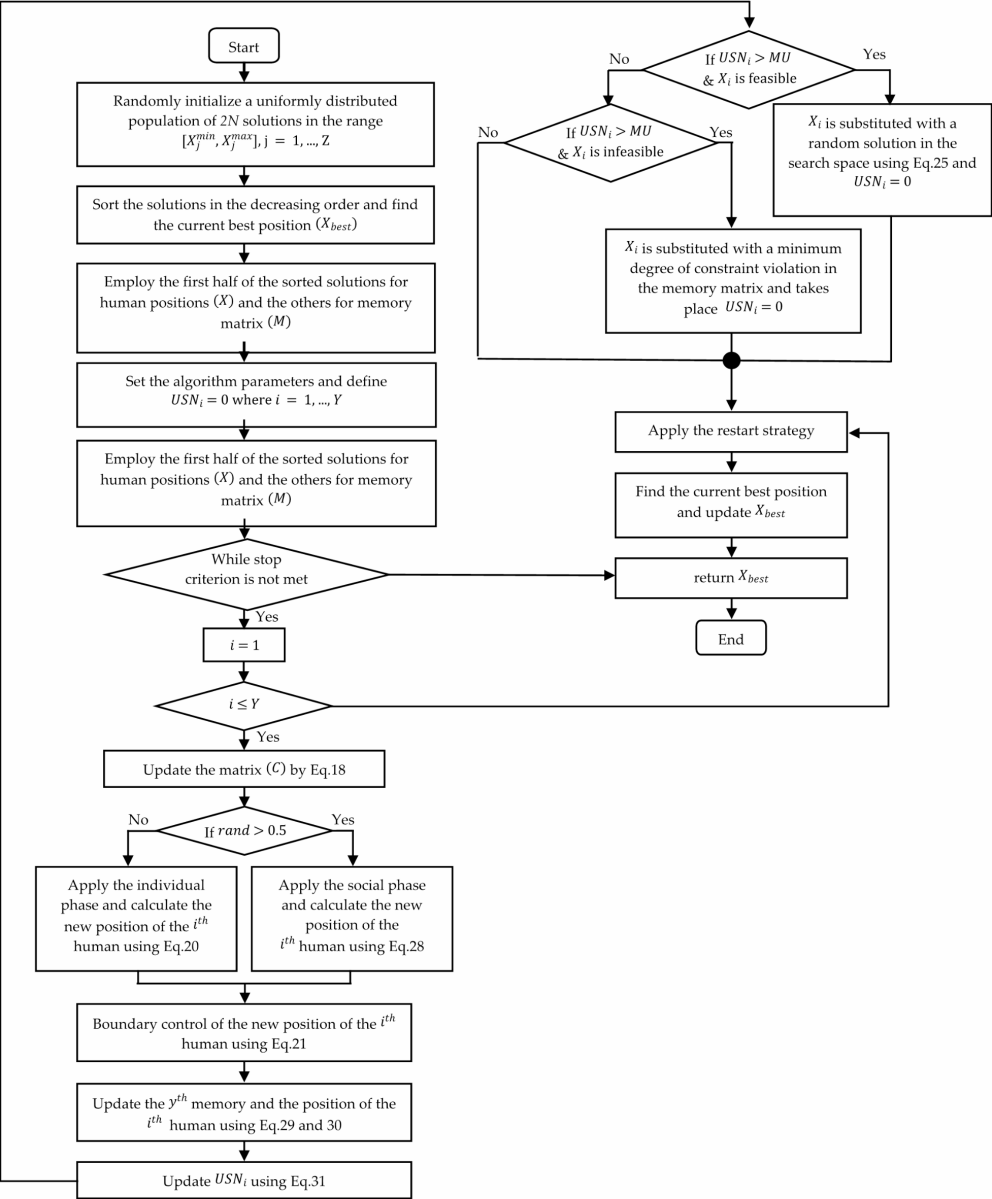
SAR method flow chart.

The SAR method flowchart is described in Fig. 1.

## Experimental results and analysis

To demonstrate the functionality of the suggested SAR algorithm, three examined scenarios based on the IEEE 30-bus, IEEE 14-bus and IEEE 57-bus test systems are conducted. The parameters of SAR method are 30 for population size, and number of evaluation is 10,000. Successful validations of the suggested SAR and other algorithms such as harmony search algorithm^51^, gradient method^5^, Artificial bee colony^52^, gravitational search algorithm^53^, particle swarm optimization^53^, Jaya algorithm^54^, enhanced genetic algorithm^55^, modified shuffle frog leaping algorithm^18^, differential evolution algorithm^46,56^, and biogeography-based optimization^47^ are performed on these systems. The cases under study are divided into four main fitness function:

### Case 1

Reducing fuel cost for generators.

### Case 2

Reducing bus voltage deviation.

### Case 3

Reducing active power losses.

### Case4

Reducing the fuel cost and the voltage deviation as a multi objective function.

MATLAB R2015b software and Intel(R) Core(TM) i7-4600U CPU @ 2.10–2.70 GHz hardware with Windows User 10 Pro and 8 GB RAM were used for the independent runs.

### Simulation results of IEEE 14 bus system

**Fig. 2:**
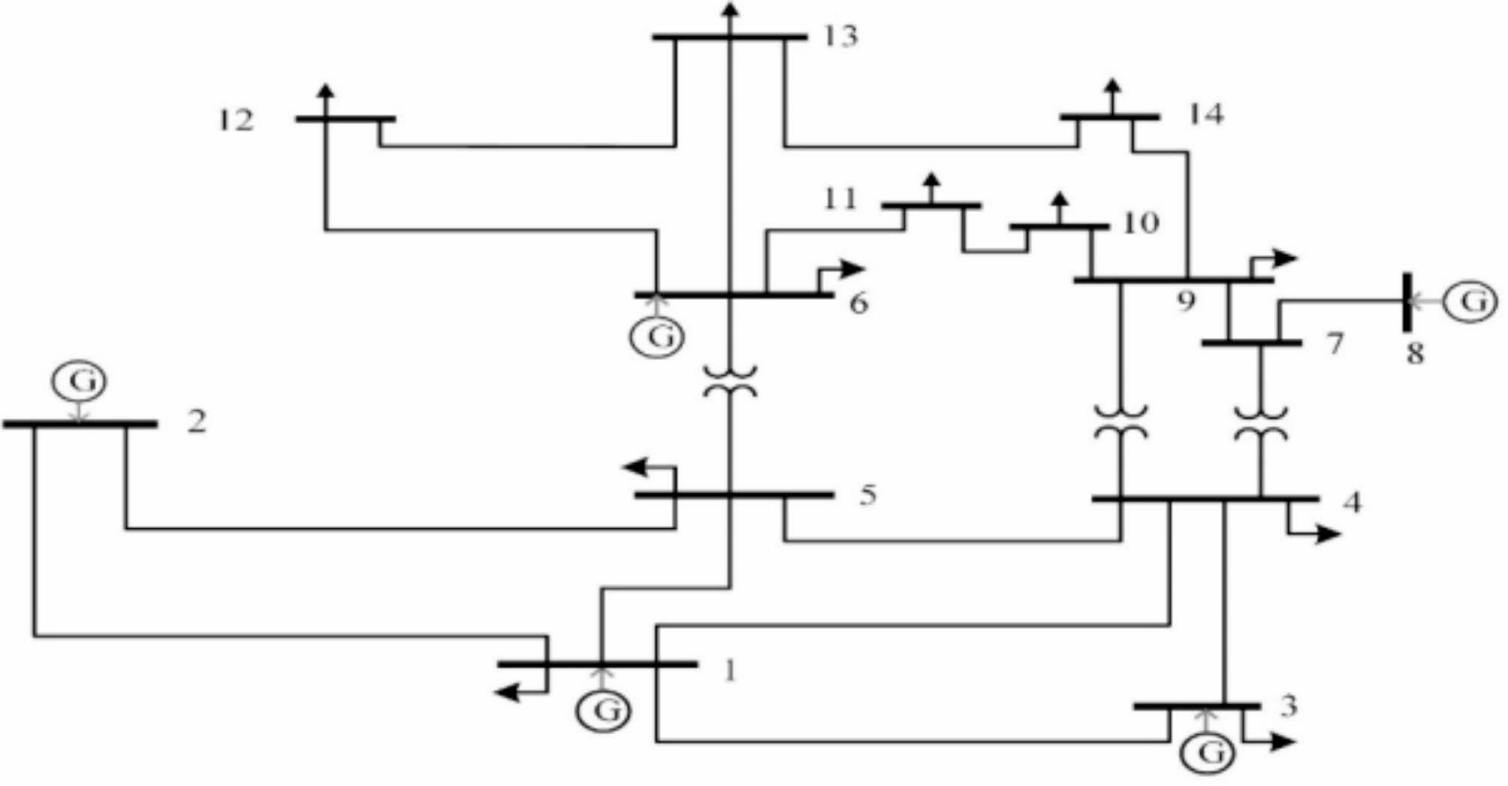
Block diagram of IEEE 14-bus system.

Figure 2 displays the IEEE 14-bus test system’s single line diagram. It has 9 load nodes and 5 generators. The system statistics and operating conditions are presented in^58^. Three regulating transformers are located in lines 5–6, 4–9, and 4–7, and five generators are located at buses 8, 6, 3, 2, and 1.

The buses’ voltage magnitudes are seen as ranging between [$\:0.95\--1.1\:p.u$]. The regulating transformers’ tap settings lie between the voltage range of [$\:0.9\--1.1\:p.u$.].

#### Results of OPFFF1 for IEEE 14-bus system

The main fitness function discussed in this subsection is minimizing the total cost of fuel. Table 1 displays the best configurations for OPF based on SAR technique for IEEE 14-bus system based on OPFFF1, that include the optimum fuel cost (objective function), voltage deviations, power loss, and control parameters settings.

**Table 1:** Best parameters solution of OPF problem extracted from SAR method based on OPFFF1 for IEEE 14-bus system.

Parameters	units	Max limit	Min limit	Best value
P1	MW	200	0	196.675310292454
P2	MW	140	0	36.9230673923451
P3	MW	100	0	27.2980711096558
P6	MW	100	0	1.53675137934193e-06
P8	MW	100	0	6.85149614064009
V1	$$\:p.u$$	1.1	0.95	1.09999999554954
V2	$$\:p.u$$	1.1	0.95	1.08237227820989
V3	$$\:p.u$$	1.1	0.95	1.05819172799031
V6	$$\:p.u$$	1.1	0.95	1.08492218633077
V8	$$\:p.u$$	1.1	0.95	1.09999996356976
T4-7	--	1.1	0.9	0.985358159802891
T4-9	--	1.1	0.9	1.09999978473857
T5-6	--	1.1	0.9	1.00590965534685
QC14	MVAR	5	0	4.99988370305641
Fitness function (Total cost of fuel in ($/h))			8051.12225602148	
Power losses in MW			8.75794646073728	
Voltage deviation			0.645803229801324	

**Fig. 3:**
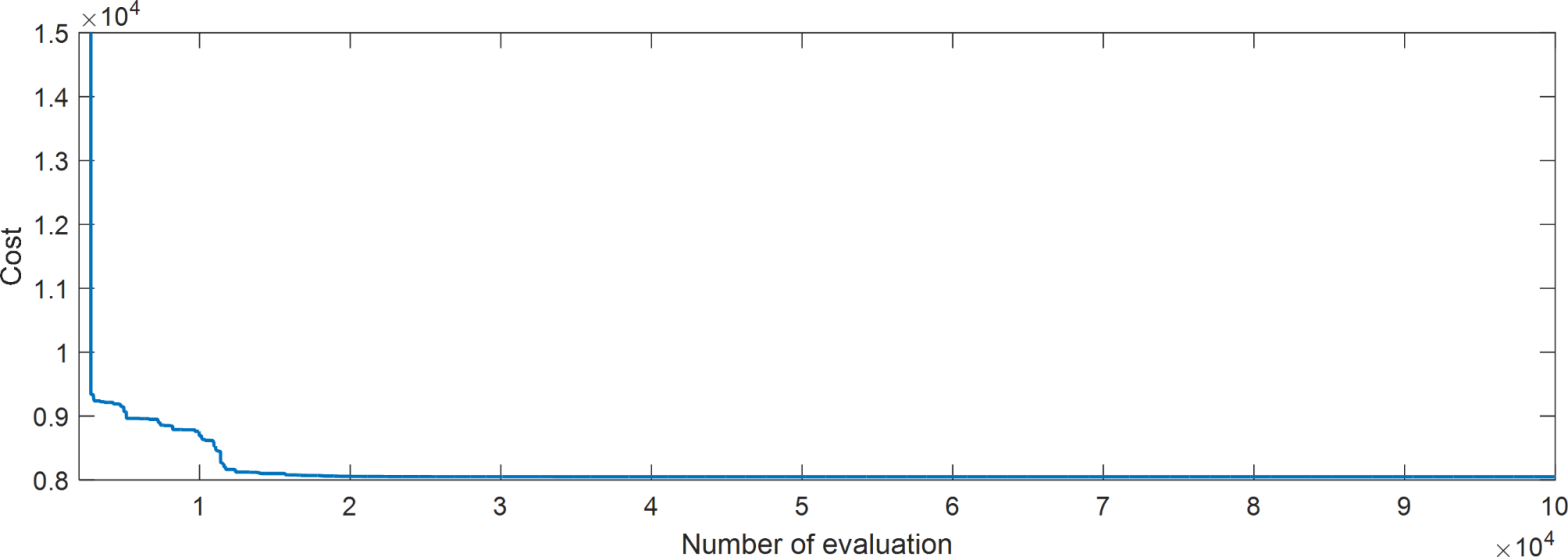
Convergence curve of SAR method based on OPFFF1 for IEEE 14-bus system.

The proposed SAR algorithm is composed with several methods such as practical swarm optimizer (PSO), Moth flam optimizer (MFO), whale and moth flam optimizer (WMFO), grey wolf optimizer (GWO), and cheap optimization algorithm (ChOA). Table 2 displays the best fitness function for OPF based on SAR technique and all comparator methods for OPFFF1, also the power loss is including in this table. Figure 3 explains the convergence curve of SAR method to reach the best solution of OPFFF1 for IEEE 14-bus system. Based on the recorded data in Table 2; the SAR algorithm achieves the best objective function (total fuel cost) compared with the other methods. The value of fitness function based on SAR technique is equal to 8051.12225602148 $/h. The order of algorithms based on the best fuel cost is SAR, MFO, WMFO, PSO, GWO and ChOA. The order of algorithms based on the power losses is SAR, PSO, WMFO, MFO, GWO, and ChOA. Hence the proposed SAR method has superior performance over all methods applied in this work for the OPFFF1 of IEEE 14-bus system.

**Table 2:** Comparison between SAR method and other methods based on OPFFF1 for IEEE 14-bus system.

Algorithm	Fuel cost	Power losses
SAR	8051.12225602148	8.75794646073728
PSO^58^	8095.642	9.209
MFO^58^	8078.659	9.223
WMFO^58^	8078.679	9.221
GWO^58^	8100.988	9.648
ChOA^58^	8142.158	10.168

#### Results of OPFFF2 for IEEE 14-bus system

The main fitness function discussed in this subsection is minimizing the total cost of fuel. Table 3 displays the best configurations for OPF based on SAR technique for IEEE 14-bus system based on OPFFF2, that include the fuel cost, the optimum voltage deviations (objective function), power loss.

**Table 3:** Best parameters solution of OPF problem extracted from SAR method based on OPFFF2 for IEEE 14-bus system.

Parameters	Units	Best value
P1	MW	2.93397270953455
P2	MW	115.306330604569
P3	MW	4.48038946406919
P6	MW	40.3951253628995
P8	MW	99.8941703336029
V1	$$\:p.u$$	0.999670186231509
V2	$$\:p.u$$	1.00038375267525
V3	$$\:p.u$$	0.999856284023542
V6	$$\:p.u$$	1.00874226264043
V8	$$\:p.u$$	1.02654389444729
T4-7	--	1.01393163540060
T4-9	--	1.07013425188454
T5-6	--	1.06253669273422
QC14	MVAR	4.98510379628612
Total cost of fuel in ($/h)		11596.1581994691
Power losses in MW		4.00743987612710
Fitness function (Voltage deviation)		0.0357680148269292

**Fig. 4:**
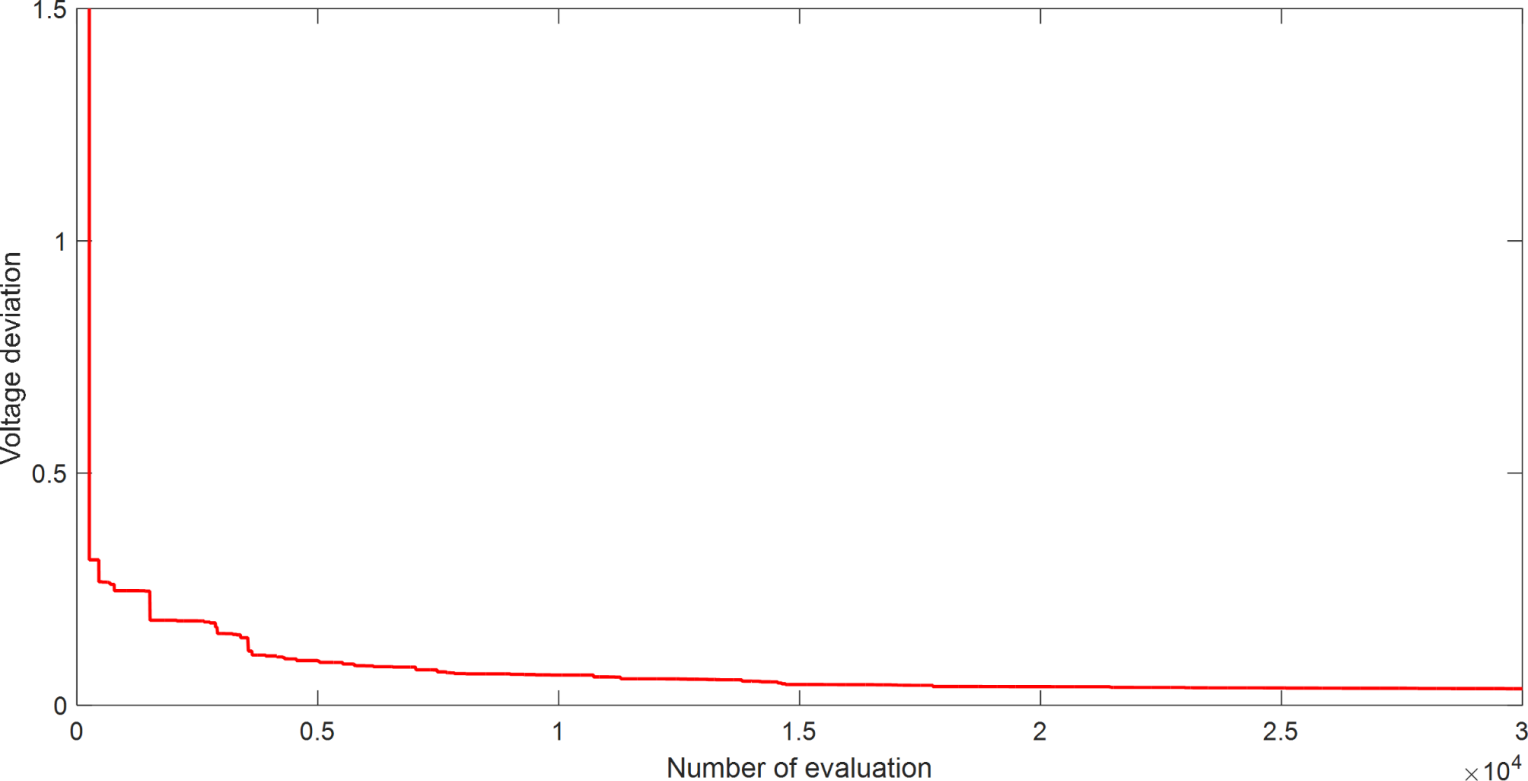
Convergence curve of SAR method based on OPFFF2 for IEEE 14-bus system.

Figure 4 explains the convergence curve of SAR method to reach the best solution of OPFFF2 for IEEE 14-bus system. Based on the recorded data in Table 3; the SAR algorithm achieves the best objective function (voltage deviation) with value 0.035768014826929 compared with its value as in case OPFFF1 equal to 0.645803229801324. Hence the proposed SAR method has superior performance in this work for the OPFFF2 of IEEE 14-bus system.

#### Results of OPFFF3 for IEEE 14-bus system

The main fitness function discussed in this subsection is minimizing the total cost of fuel. Table 4 displays the best configurations for OPF based on SAR technique for IEEE 14-bus system based on OPFFF3, that include the optimum fuel cost, voltage deviations (objective function), power loss.

**Table 4:** Best parameters solution of OPF problem extracted from SAR method based on OPFFF3 for IEEE 14-bus system.

Parameters	Units	Best value
P1	MW	0.244656771401016
P2	MW	21.6410969619936
P3	MW	94.4225728850474
P6	MW	46.7021482615347
P8	MW	96.4589227187924
V1	$$\:p.u$$	1.04436783036729
V2	$$\:p.u$$	1.04415997450881
V3	$$\:p.u$$	1.04394618915954
V6	$$\:p.u$$	1.05901961788393
V8	$$\:p.u$$	1.09999618380480
T4-7	--	1.00214096595492
T4-9	--	1.09994598905619
T5-6	--	1.00499140591328
QC14	MVAR	4.99802255741571
Total cost of fuel in ($/h)		10262.1580412576
Fitness function (Power losses in MW)		0.459733441487247
Voltage deviation		0.419330368553937

**Fig. 5:**
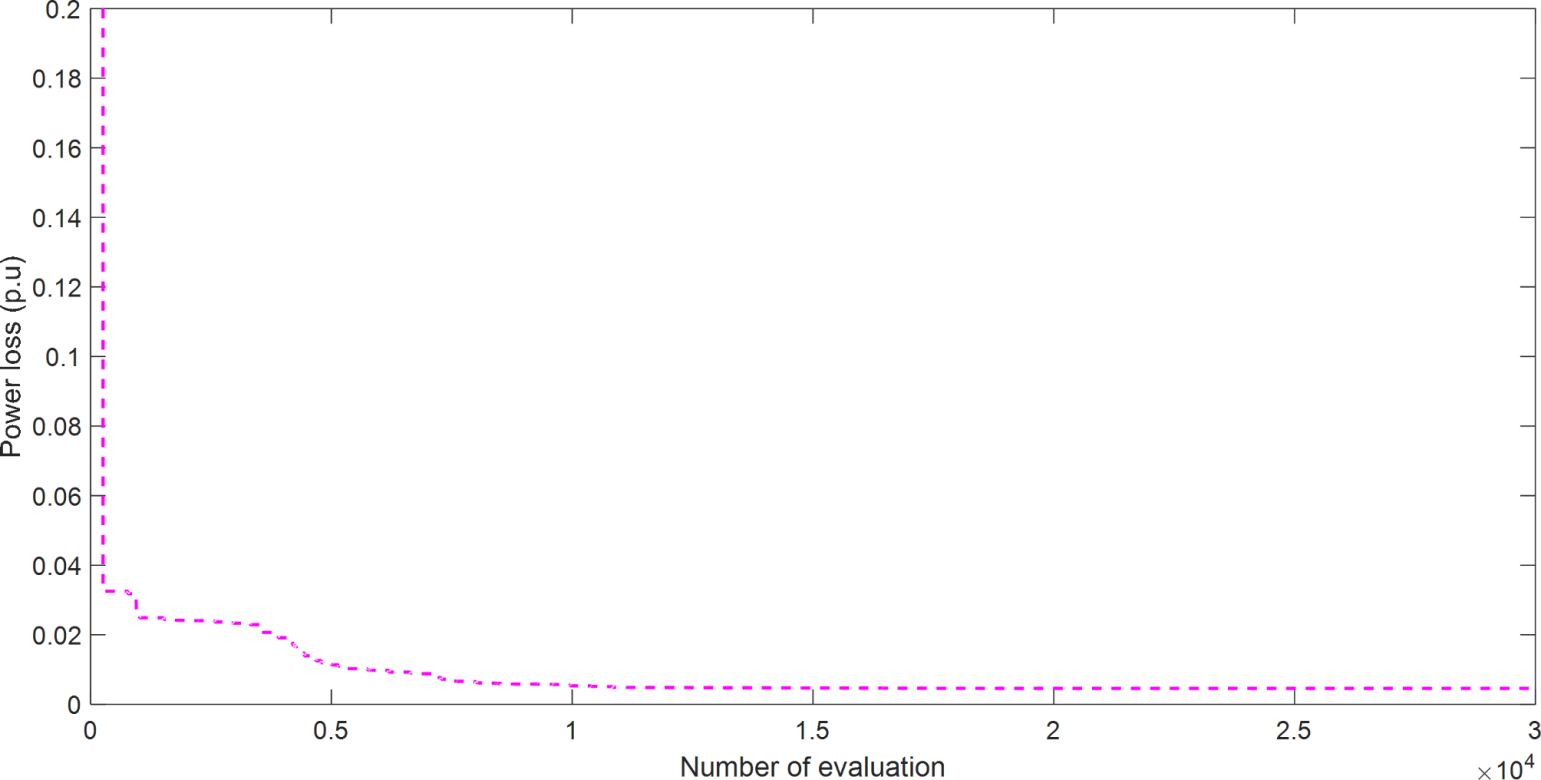
Convergence curve of SAR method based on OPFFF3 for IEEE 14-bus system.

Figure 5 explains the convergence curve of SAR method to reach to the best solution of OPFFF3 for IEEE 14-bus system. Based on the recorded data in Table 4; the SAR algorithm achieve the best objective function (power loss) with value 0.459733441487247 compared with its value as in case OPFFF1 equal to 8.75794646073728. Hence the proposed SAR method has superior performance in this work for the OPFFF3 of IEEE 14-bus system.

#### Results of OPFFF4 for IEEE 14-bus system

The main fitness function discussed in this subsection is minimizing the total cost of fuel. Table 5 displays the best configurations for OPF based on SAR technique for IEEE 14-bus system based on OPFFF4, that include the optimum fuel cost, voltage deviations (objective function), power loss.

**Table 5:** Best parameters solution of OPF problem extracted from SAR method based on OPFFF4 for IEEE 14-bus system.

Parameters	Units	Best value
P1	MW	197.127887976055
P2	MW	36.9805125739630
P3	MW	27.5945955959562
P6	MW	0.000563735380294885
P8	MW	6.28990109376045
V1	$$\:p.u$$	1.09999958459040
V2	$$\:p.u$$	1.08049491759772
V3	$$\:p.u$$	1.04978668797757
V6	$$\:p.u$$	1.01738240151780
V8	$$\:p.u$$	1.04512711237670
T4-7	--	0.900019420961687
T4-9	--	1.08910987239498
T5-6	--	1.00676316882710
QC14	MVAR	4.99959881691876
Fitness function (Total cost of fuel in ($/h))		8059.56165483559
Power losses in MW		9.00041913011900
Fitness function (Voltage deviation)		0.115870519085356

**Fig. 6:**
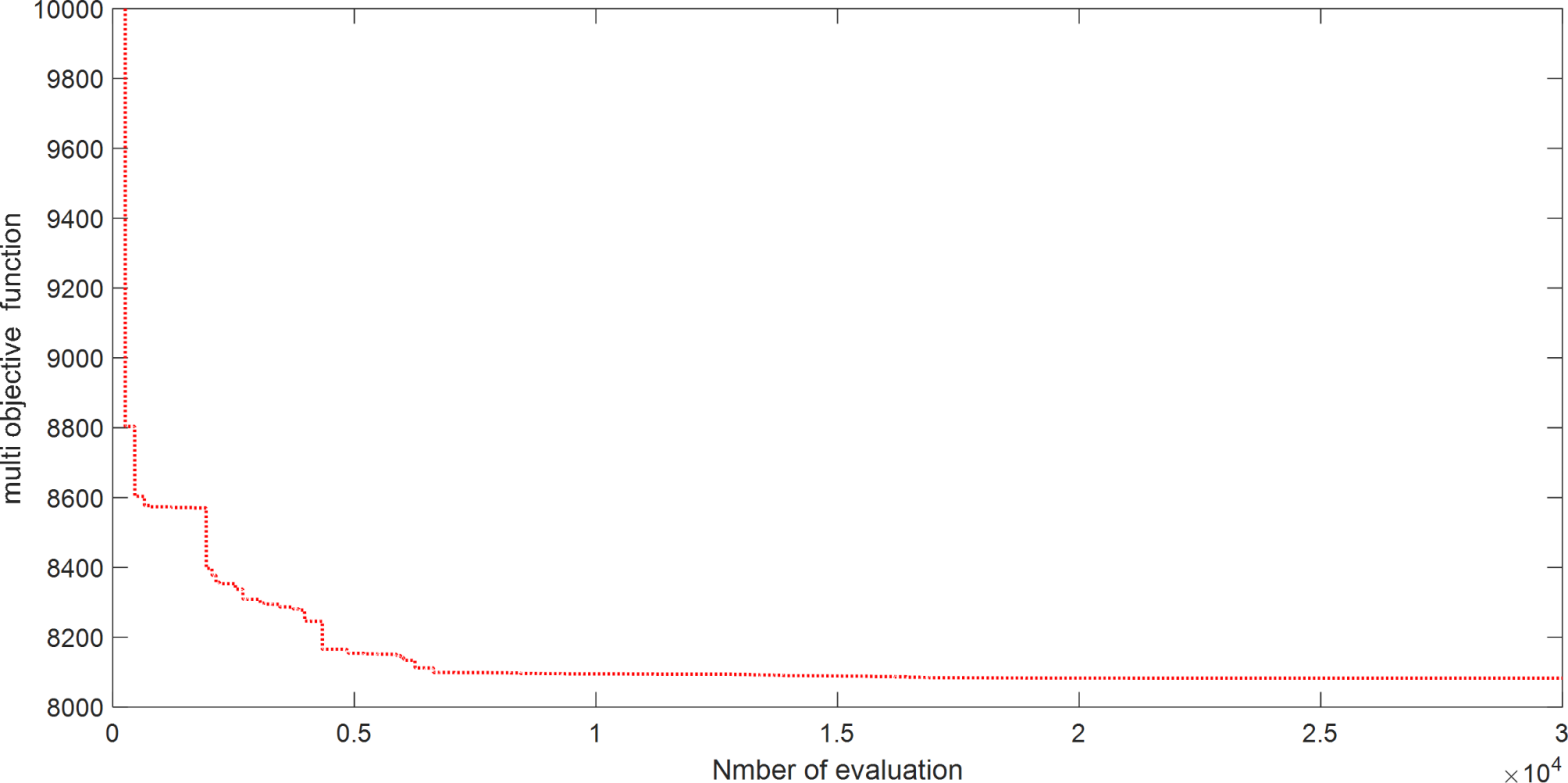
Convergence curve of SAR method based on OPFFF4 for IEEE 14-bus system.

The proposed SAR algorithm is composed with several methods such as practical swarm optimizer (PSO), Moth flam optimizer (MFO), whale and moth flam optimizer (WMFO), grey wolf optimizer (GWO), and cheap optimization algorithm (ChOA). Table 6 displays the best fitness function for OPF based on SAR technique and all comparator methods for OPFFF1, also the power loss is included in this table. Figure 6 explains the convergence curve of SAR method to reach the best solution of OPFFF4 for IEEE 14-bus system. Based on the recorded data in Table 6; the SAR algorithm achieves the best objective function (total fuel cost) compared with the other methods. The value of cost based on the fitness function extracted from SAR technique is equal to 8059.56165483559 $/h. The value of voltage deviation based on the fitness function extracted from SAR technique is equal to 0.115870519085356. The order of algorithms based on the best fuel cost is SAR, WMFO, MFO, GWO, PSO and ChOA. The order of algorithms based on the power losses is SAR, MFO, WMFO, PSO, GWO, and ChOA. Hence the proposed SAR method has superior performance over all methods applied in this work for the OPFFF4 of IEEE 14-bus system.

**Table 6:** Comparison between SAR method and other methods based on OPFFF4 for IEEE 14-bus system.

Algorithm	Fuel cost	Power losses
SAR	8059.56165483559	9.00041913011900
PSO^58^	8103.609	10.317
MFO^58^	8082.392	9.349
WMFO^58^	8082.128	9.379
GWO^58^	8100.701	10.645
ChOA^58^	8143.173	11.674

### Simulation results of IEEE 30 bus system

**Fig. 7:**
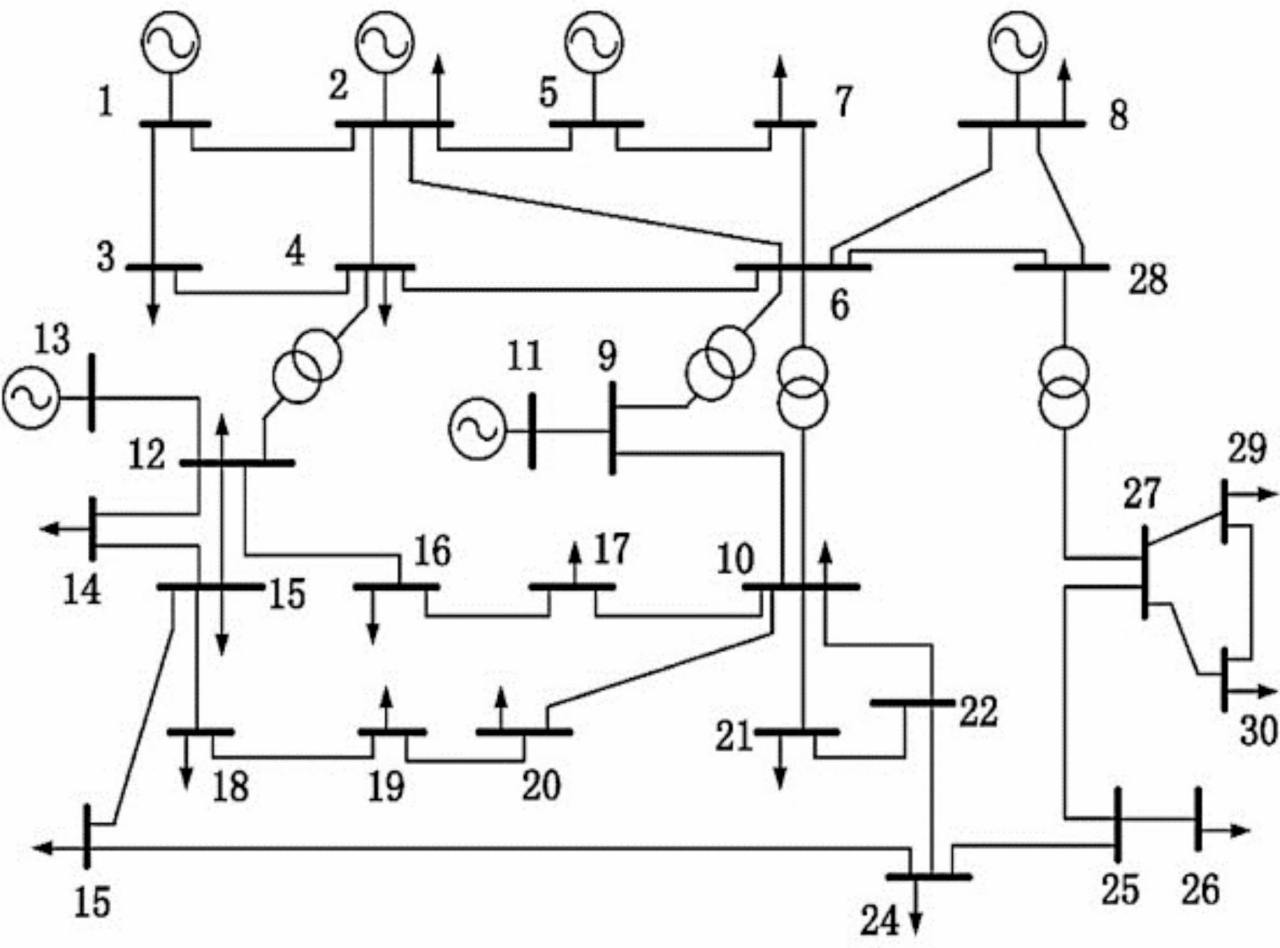
Block diagram of IEEE 30-bus system.

Figure 7 displays the IEEE 30-bus test system’s single line diagram. It has 24 load nodes and 6 generators. The system statistics and operating conditions are presented in^5,58^. Four regulating transformers are located in lines 6–10, 6–9, 27–28, and 4–12, and six generators are located at buses 13, 11, 8, 5, 2, and 1. In addition, buses 29, 24, 23, 21, 20, 17, ,15, 12, and 10 include sources of reactive power.

The buses’ voltage magnitudes are seen as ranging between [$\:0.95\--1.1\:p.u$]. The regulating transformers’ tap settings lie between the voltage range of [$\:0.9\--1.1\:p.u$.]. The MVAR shunt capacitor ratings vary between 0 and 5. Permissible operation limitations [$\:0.95\--1.05\:p.u$.] apply to the load buses. Table 7 display the generator cost curve data for the IEEE 30-bus test system.

**Table 7:** Parameters coefficient of generators for IEEE 30-bus system.

Bus	C $\:\left(\text{\$}/M{W}^{2}\right)$	b $\:\left(\text{\$}/MW\right)$	a $\:\left(\text{\$}\right)$
13	0.025	3	0
11	0.025	3	0
8	0.00834	3.25	0
5	0.0625	1	0
2	0.0175	1.75	0
1	0.00375	2	0

#### Results of OPFFF1 for IEEE 30-bus system

The main fitness function discussed in this subsection is minimizing the total cost of fuel. Table 8 displays the best configurations for OPF based on SAR technique for IEEE 30-bus system based on OPFFF1, that include the optimum fuel cost (objective function), voltage deviations, power loss, and control parameters settings.

**Table 8:** Best parameters solution of OPF problem extracted from SAR method based on OPFFF1 for IEEE 30-bus system.

Parameters	Units	Max limit	Min limit	Best value
P1	MW	200	50	178.414192084461
P2	MW	80	20	48.7329094315295
P5	MW	50	15	21.0888527342911
P8	MW	35	10	20.7472465173017
P11	MW	30	10	11.7372914792274
P13	MW	40	12	11.1611625186174
V1	$$\:p.u$$	1.1	0.95	1.13851922405740
V2	$$\:p.u$$	1.1	0.95	1.11949682757984
V5	$$\:p.u$$	1.1	0.95	1.08868437369405
V8	$$\:p.u$$	1.1	0.95	1.09589696926968
V11	$$\:p.u$$	1.1	0.95	1.04861211036109
V13	$$\:p.u$$	1.1	0.95	1.11381890405985
T11	--	1.1	0.9	0.937437520030738
T12	--	1.1	0.9	1.03475077231005
T15	--	1.1	0.9	0.983194859186078
T36	--	1.1	0.9	1.01392751240078
QC10	MVAR	5	0	2.15004331752991
QC12	MVAR	5	0	6.71692479945985
QC15	MVAR	5	0	2.85869652401398
QC17	MVAR	5	0	6.75663842039920
QC20	MVAR	5	0	2.82674116255692
QC21	MVAR	5	0	11.7303296097117
QC23	MVAR	5	0	2.59736550237822
QC24	MVAR	5	0	1.24145613293041
QC29	MVAR	5	0	3.14346861231495
Fitness function (Total cost of fuel in ($/h))			798.197578585806	
Power losses in MW			8.49155243457396	
Voltage deviation			2.07934784296484	

**Fig. 8:**
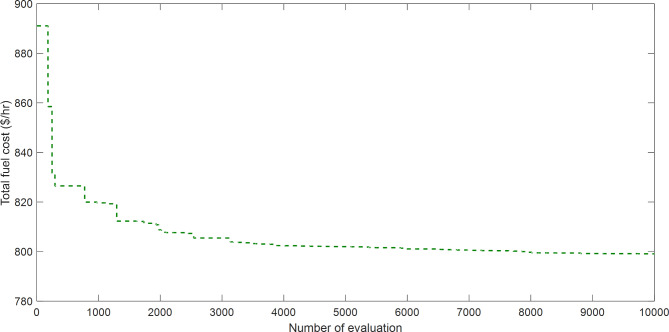
Convergence curve of SAR method based on OPFFF1 for IEEE 30-bus system.

The proposed SAR algorithm is composited with several methods such as gradient method (GM), adaptive genetic algorithm (AGA), Artificial bee colony (ABC), gravitational search algorithm (GSA), particle swarm optimization (PSO), Jaya algorithm (JAYA), modified shuffle frog leaping algorithm (MSFLA), and differential evolution algorithm (DE). Table 9 displays the best fitness function for OPF based on SAR technique and all comparator methods for OPFFF1, also the power loss is including in this table. Figure 8 explains the convergence curve of SAR method to reach to the best solution of OPFFF1. Based on the recorded data in Table 9; the SAR algorithm achieves the best objective function (total fuel cost) compared with the other methods. The value of fitness function based on SAR technique is equal to 798.197578585806 $/h. The order of algorithms based on the fuel cost is SAR, MSCA, AGA-POP, SCA, JAYA, GSA and PSO, WMFO, ABC, MSFLA, DE, GM, GWO and ChOA. The order of algorithms based on the power losses is SAR, MSCA, AGA-POP, ABC, GSA and PSO, SCA, WMFO, JAYA, GWO, DE, MSFLA, GM and ChOA. Hence the proposed SAR method has superior performance over all methods applied in this work for the OPFFF1 of IEEE 30-bus system.

**Table 9:** Comparison between SAR method and others methods based on OPFFF1 for IEEE 30-bus system.

Algorithm	Fuel cost	Power losses
SAR	798.197578585806	8.49155243457396
GM^5^	804.853	10.486
MSFLA^18^	802.287	9.6991
ABC^52^	800.66	9.0328
JAYA^54^	800.4794	9.06481
GSA and PSO^53^	800.49589	9.0339
AGA-POP^13^	799.8441	8.9166
DE^46^	802.394	9.466
SCA^5^	800.1018	9.0633
MSCA^5^	799.31	8.7327
GWO^58^	803.375	9.082
ChOA^58^	818.495	11.236
WMFO^58^	800.603	9.066

#### Results of OPFFF2 for IEEE 30-bus system

The main fitness function discussed in this subsection is minimizing the voltage deviation (OPFFF2). Table 10 displays the best configurations for OPF based on SAR technique for OPFFF2, that include voltage deviations (objective function), power loss, total fuel cost, and control parameter settings.

**Table 10:** Best parameters solution of OPF problem extracted from SAR method based on OPFFF2 for IEEE 30-bus system.

Parameters	units	Max limit	Min limit	Best value
P1	MW	200	50	55.0231402967134
P2	MW	80	20	55.6114263066869
P5	MW	50	15	56.5433457728002
P8	MW	35	10	61.0616642165355
P11	MW	30	10	18.9070554552523
P13	MW	40	12	39.5951079306953
V1	$$\:p.u$$	1.1	0.95	1.00564529846452
V2	$$\:p.u$$	1.1	0.95	1.00761927284532
V5	$$\:p.u$$	1.1	0.95	1.00717581492474
V8	$$\:p.u$$	1.1	0.95	1.01834251696240
V11	$$\:p.u$$	1.1	0.95	0.953134539424718
V13	$$\:p.u$$	1.1	0.95	0.998991760153556
T11	--	1.1	0.9	1.02434509501019
T12	--	1.1	0.9	0.976683778076507
T15	--	1.1	0.9	1.02019960465305
T36	--	1.1	0.9	1.03594769876269
QC10	MVAR	5	0	1.03015651450185
QC12	MVAR	5	0	3.90122355278198
QC15	MVAR	5	0	4.13913068948280
QC17	MVAR	5	0	4.11862821323234
QC20	MVAR	5	0	6.89924453121801
QC21	MVAR	5	0	7.17033696271725
QC23	MVAR	5	0	4.15135670017700
QC24	MVAR	5	0	7.22684077674516
QC29	MVAR	5	0	2.05394862283341
Fitness function (Voltage deviation)			0.0978069572088536	
Power losses in MW			3.35090200543612	
Total cost of fuel in ($/h)			982.389875241927	

**Fig. 9:**
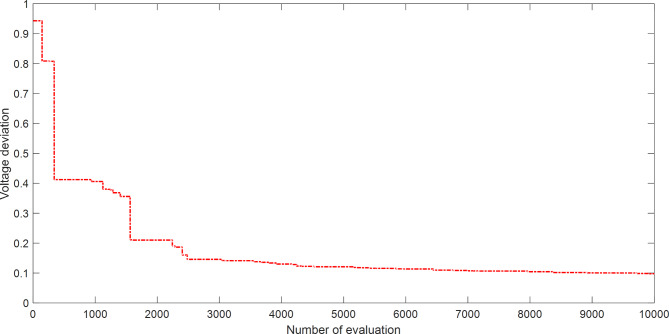
Convergence curve of SAR method based on OPFFF2 for IEEE 30-bus system.

The proposed SAR algorithm is composited with several methods such as harmony search algorithm (HS)^51^, biogeography-based optimization (BBO)^57^, Jaya algorithm (JAYA)^54^, sine cosine algorithm (SCA), and its modification^5^ for objective function of OPFFF2. Table 11 displays the best fitness function for OPF based on SAR technique and all comparator methods for OPFFF2, that include real power loss and voltage deviation. Figure 9 explains the convergence curve of SAR method to reach the best solution of OPFFF2 for IEEE 30-bus system. Based on the recorded data in Table 11; the SAR algorithm achieves the best objective function (voltage deviation) compared with the other methods. The value of fitness function based on SAR technique is equal to 0.0978069572088536. The order of methods based on the fuel cost is SAR, BBO, HS, MSCA, SCA, and JAYA. The order of algorithms based on the power losses is SAR, HS, BBO, MSCA, JAYA, and SCA. Hence the proposed SAR method has superior performance over all methods applied in this work of OPFFF2 for IEEE 30-bus system.

**Table 11:** Comparison between SAR method and other methods based on OPFFF2 for IEEE 30-bus system.

Algorithm	Voltage deviation	Power loss
SAR	0.0978069572088536	3.35090200543612
HS^51^	0.1006	4.3244
BBO^57^	0.09803	4.95
Jaya^54^	0.1243	7.884
SCA^5^	0.1082	8.5031
MSCA^5^	0.1031	7.0828

#### Results of OPFFF3 for IEEE 30-bus system

The main fitness function discussed in this subsection is minimizing the real power losses (OPFFF3). Table 12 displays the best configurations for OPF based on SAR technique for OPFFF3, that include power loss (objective function), voltage deviations, total fuel cost, and control parameter settings.

**Table 12:** Best parameters solution of OPF problem extracted from SAR method based on OPFFF3 for IEEE 30-bus system.

Parameters	units	Max limit	Min limit	Best value
P1	MW	200	50	50.4237130469997
P2	MW	80	20	49.0161819773966
P5	MW	50	15	49.9583643572817
P8	MW	35	10	40.6231402443484
P11	MW	30	10	51.3136576960585
P13	MW	40	12	44.7682582266897
V1	$$\:p.u$$	1.1	0.95	1.06469526713309
V2	$$\:p.u$$	1.1	0.95	1.05542297903514
V5	$$\:p.u$$	1.1	0.95	1.04086718903500
V8	$$\:p.u$$	1.1	0.95	1.04576864743066
V11	$$\:p.u$$	1.1	0.95	1.03909969148273
V13	$$\:p.u$$	1.1	0.95	1.09616332432340
T11	--	1.1	0.9	0.964034752738131
T12	--	1.1	0.9	1.09543643378739
T15	--	1.1	0.9	1.00152249176317
T36	--	1.1	0.9	0.990276943033739
QC10	MVAR	5	0	7.32792717698242
QC12	MVAR	5	0	5.66401527789931
QC15	MVAR	5	0	3.27601610944955
QC17	MVAR	5	0	3.29185999758433
QC20	MVAR	5	0	3.73055818040562
QC21	MVAR	5	0	2.09058796685088
QC23	MVAR	5	0	0.906994812587805
QC24	MVAR	5	0	6.88316617591059
QC29	MVAR	5	0	5.64246405374752
Power losses in MW			2.71286428848434	
Voltage deviation			1.34433424745398	
Total cost of fuel in ($/h)			994.119978261098	

**Fig. 10:**
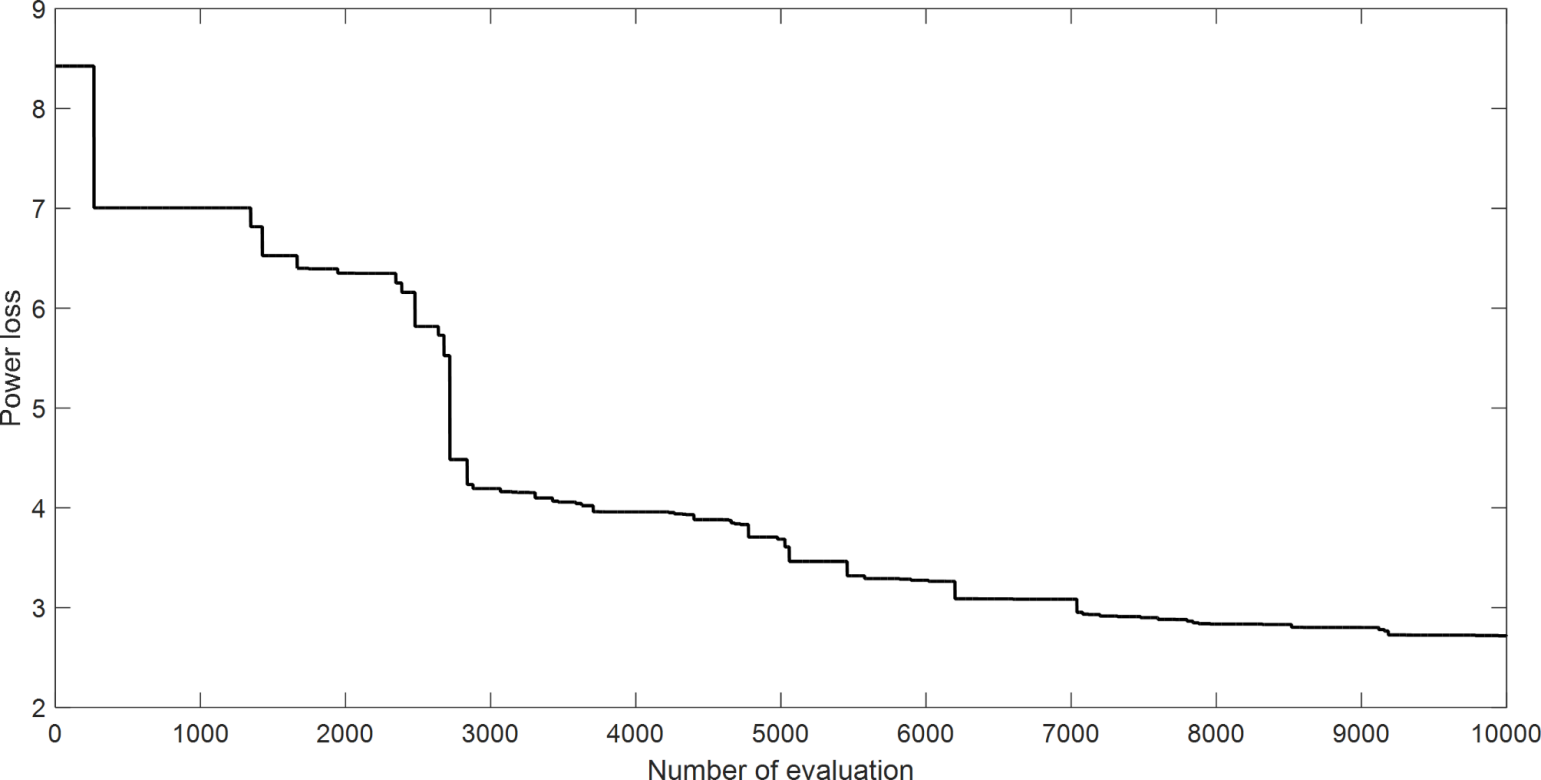
Convergence curve of SAR method based on OPFFF3 for IEEE 30-bus system.

The proposed SAR algorithm is composed of several methods such as harmony search algorithm^34^, Artificial bee colony^52^, Jaya algorithm^54^, enhanced genetic algorithm^55^, modified sine cosine algorithm^5^. Table 13 displays the best fitness function for OPF based on SAR technique and all comparator methods for OPFFF3, that include power loss (objective function), and voltage deviation. Figure 10 explains the convergence curve of SAR method to reach the best solution of OPFFF3. Based on the recorded data in Table 13; the SAR algorithm achieves the best objective function (power losses) compared with the other methods. The value of fitness function based on SAR technique is equal to 2.71286428848434 MW. The order of methods based on the power losses is SAR, MSCA, HS, JAYA, ABC, and EGA. Hence the proposed SAR method has superior performance over all methods applied in this work for OPFFF3 of IEEE 30-bus system.

**Table 13:** Comparison between SAR method and other methods based on OPFFF3 for IEEE 30-bus system.

Algorithm	Power losses (MW)
SAR	2.71286428848434
MSCA^5^	2.9334
HS^51^	2.9678
ABC^52^	3.1078
JAYA^54^	3.1035
EGA^55^	3.2008

#### Results of OPFFF4 for IEEE 30-bus system

The main fitness function discussed in this subsection is minimizing the real power losses (OPFFF4). Table 14 displays the best configurations for OPF based on SAR technique for OPFFF4, that include the value of multi-objective function, the power loss, voltage deviations, total fuel cost, and control parameter settings.

**Table 14:** Best parameters solution of OPF problem extracted from SAR method based on OPFFF4 for IEEE 30-bus system.

Parameters	units	Max limit	Min limit	Best value
P1	W	200	50	172.250098007987
P2	W	80	20	48.0911958995797
P5	W	50	15	21.6026540840626
P8	W	35	10	25.8023463918586
P11	W	30	10	12.7981814019926
P13	W	40	12	13.3634370125359
V1	$$\:p.u$$	1.1	0.95	1.01673965603912
V2	$$\:p.u$$	1.1	0.95	0.999947291366025
V5	$$\:p.u$$	1.1	0.95	1.00003528109290
V8	$$\:p.u$$	1.1	0.95	1.01594171486256
V11	$$\:p.u$$	1.1	0.95	1.00000199092240
V13	$$\:p.u$$	1.1	0.95	1.00001055743770
T11	--	1.1	0.9	0.983530250128821
T12	--	1.1	0.9	1.00208650914258
T15	--	1.1	0.9	1.02529894499459
T36	--	1.1	0.9	1.03288913034679
QC10	MVAR	5	0	4.79747617073683
QC12	MVAR	5	0	4.94039445156757
QC15	MVAR	5	0	4.98564852976735
QC17	MVAR	5	0	3.25278591814e-05
QC20	MVAR	5	0	4.99999522745882
QC21	MVAR	5	0	4.06666047293714
QC23	MVAR	5	0	4.99583992228270
QC24	MVAR	5	0	4.98277516683647
QC9	MVAR	5	0	3.46321406787813
Multi-objective fitness function			834.762531525182	
Total cost of fuel in ($/h)			807.620125852399	
Voltage deviation			0.102930119375242	
Power losses in MW			10.5166452462514	

**Fig. 11:**
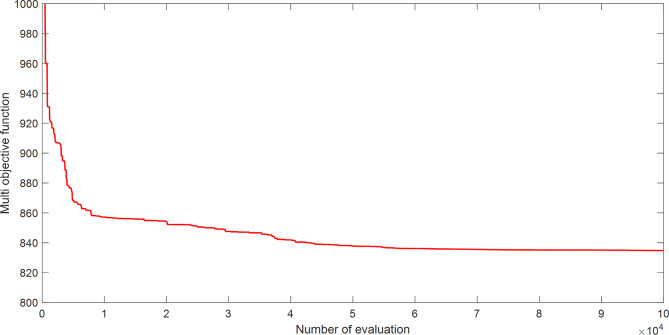
Convergence curve of SAR method based on OPFFF4 for IEEE 30-bus system.

The proposed SAR algorithm is composed of several methods such as practical swarm optimizer (PSO), moth flam optimizer (MFO), grey wolf optimizer (GWO), and cheap optimization algorithm (ChOA). Table 15 displays the best fitness function for OPF based on SAR technique and all comparator methods for OPFFF4 of IEEE 30-bus system, also the power loss is including in this table. Figure 11 explains the convergence curve of SAR method to reach to the best solution of OPFFF4 for IEEE 30-bus system. Based on the recorded data in Table 15, the SAR algorithm achieves the best objective function (total fuel cost) compared with the other methods. The value of cost based on the fitness function extracted from SAR technique is equal to 807.620125852399 $/h. The value of voltage deviation based on the fitness function extracted from SAR technique is equal to 0.102930119375242. The order of algorithms based on the best fuel cost is SAR, GWO, MFO, PSO and ChOA. The order of algorithms based on the best voltage deviation are SAR, MFO, GWO, PSO and ChOA. Hence the proposed SAR method has superior performance over all methods applied in this work for the OPFFF4 of IEEE 30-bus system.

**Table 15:** Comparison between SAR method and other methods based on OPFFF4 for IEEE 30-bus system.

Algorithm	Fuel cost	Voltage deviation	Power losses
SAR	807.620125852399	0.102930119375242	10.5166452462514
PSO	810.931	0.241	10.151
GWO	807.675	0.156	9.908
MFO	810.816	0.191	10.496
ChOA	813.642	0.375	8.357

### Simulation results of IEEE 57-bus system

**Fig. 12:**
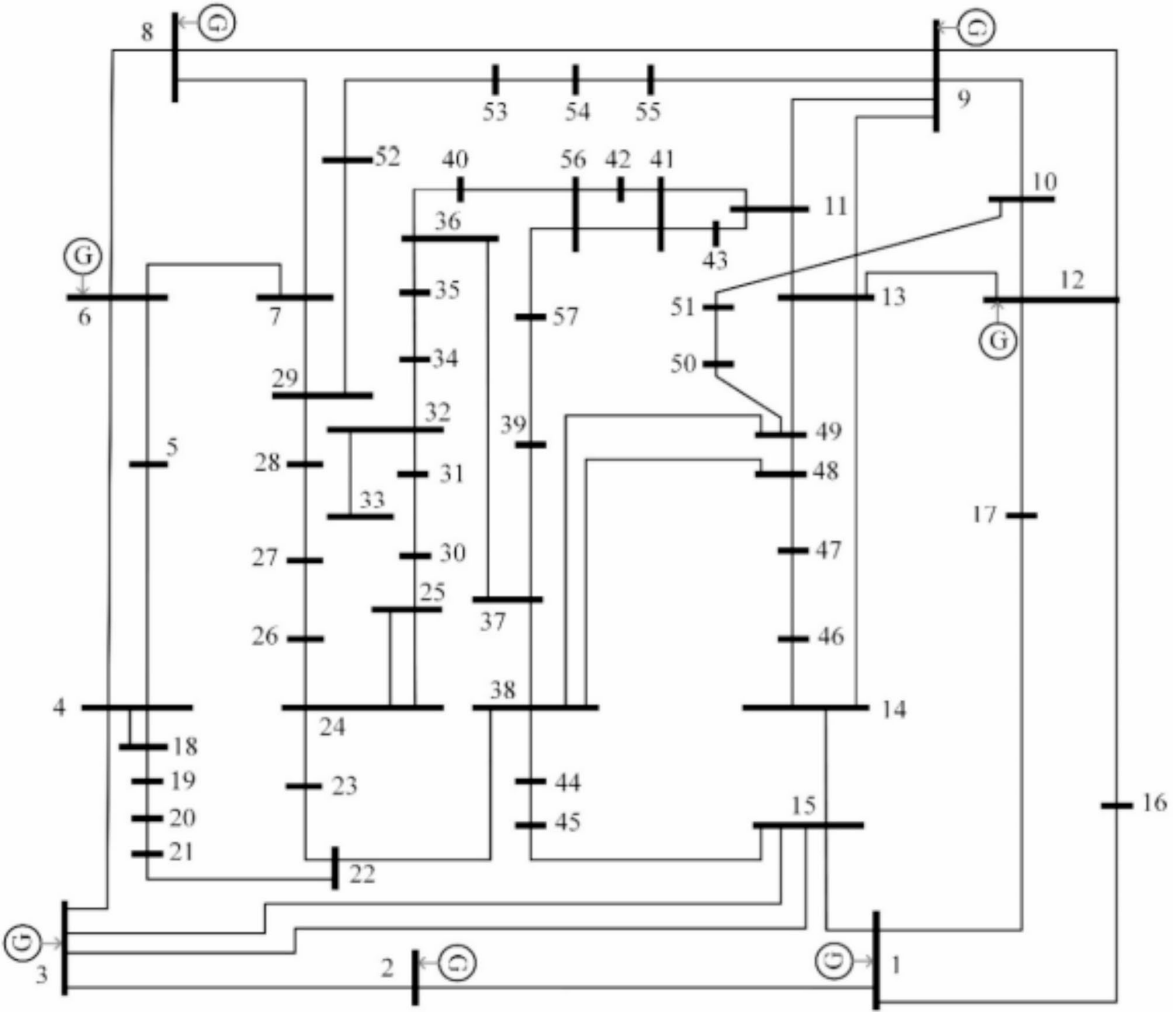
Block diagram of IEEE 57-bus system.

Figure 12 displays the IEEE 57-bus test system’s single line diagram^58^. It has 50 load nodes and 7 generators. seventeen regulating transformers are in lines 4–18, 20–21, 24–25, 24–26, 7–29, 32–34, 11–41, 15–54, 14–46, 10–51, 13–49, 11–43, 40–56, 39–57 and 9–55, and seven generators are located at buses 12, 9, 8, 6, 3, 2, and 1. In addition, buses 53, 25 and 18 include sources of reactive power. The buses’ voltage magnitudes are seen as ranging between [$\:0.95\--1.1\:p.u$]. The regulating transformers’ tap settings lie between the voltage range of [$\:0.9\--1.1\:p.u$.]. The MVAR shunt capacitor ratings vary between 0 and 30 MVAR. Permissible operation limitations [$\:0.95\--1.05\:p.u$.] apply to the load buses.

#### Results of OPFFF1 for IEEE 57-bus system

The main fitness function discussed in this subsection is minimizing the total cost of fuel. Table 16 displays the best configurations for OPF based on SAR technique for IEEE 57-bus system based on OPFFF1, that include the optimum fuel cost (objective function), voltage deviations, power loss, and control parameters settings.

**Table 16:** Best parameters solution of OPF problem extracted from SAR method based on OPFFF1 for IEEE 57-bus system.

Parameters	Units	Best value
P1	MW	6.00840125151341
P2	MW	51.5023736273341
P3	MW	132.143966797945
P6	MW	66.9564451237570
P8	MW	383.811124055354
P9	MW	80.3378444397889
P12	MW	370.745389656906
VG1	$$\:p.u$$	1.01733624166360
VG2	$$\:p.u$$	1.04137048038009
VG3	$$\:p.u$$	0.977473433274265
VG6	$$\:p.u$$	1.05510357735388
VG8	$$\:p.u$$	1.09604070411900
VG9	$$\:p.u$$	0.978159884440329
VG12	$$\:p.u$$	0.989031144748093
T(4–18)	--	0.903509898087040
T(4–18)	--	1.08335005955890
T(21 − 20)	--	0.988636319330648
T(24–25)	--	0.963710995235425
T(24–25)	--	0.975008831434853
T(24–26)	--	1.06101777242923
T(7–29)	--	0.965063748627241
T(34 − 32)	--	1.00659660019694
T(11–41)	--	1.04306152548546
T(15–45)	--	0.908049360191816
T(14–46)	--	0.942731489012128
T(10–51)	--	0.963699553605685
T(13–49)	--	0.968865924185937
T(11–43)	--	1.08547643551808
T(40–56)	--	1.07899630299445
T(39–57)	--	0.987235881500356
T(9–55)	--	0.951257830325875
QC18	MVAR	5.90570466498343
QC25	MVAR	1.67558616289805
QC53	MVAR	2.87542226410348
Fitness function (Total cost of fuel in ($/h))		38017.7691758245
Power losses in MW		21.5490022790274
Voltage deviation		1.93753027811899

**Fig. 13:**
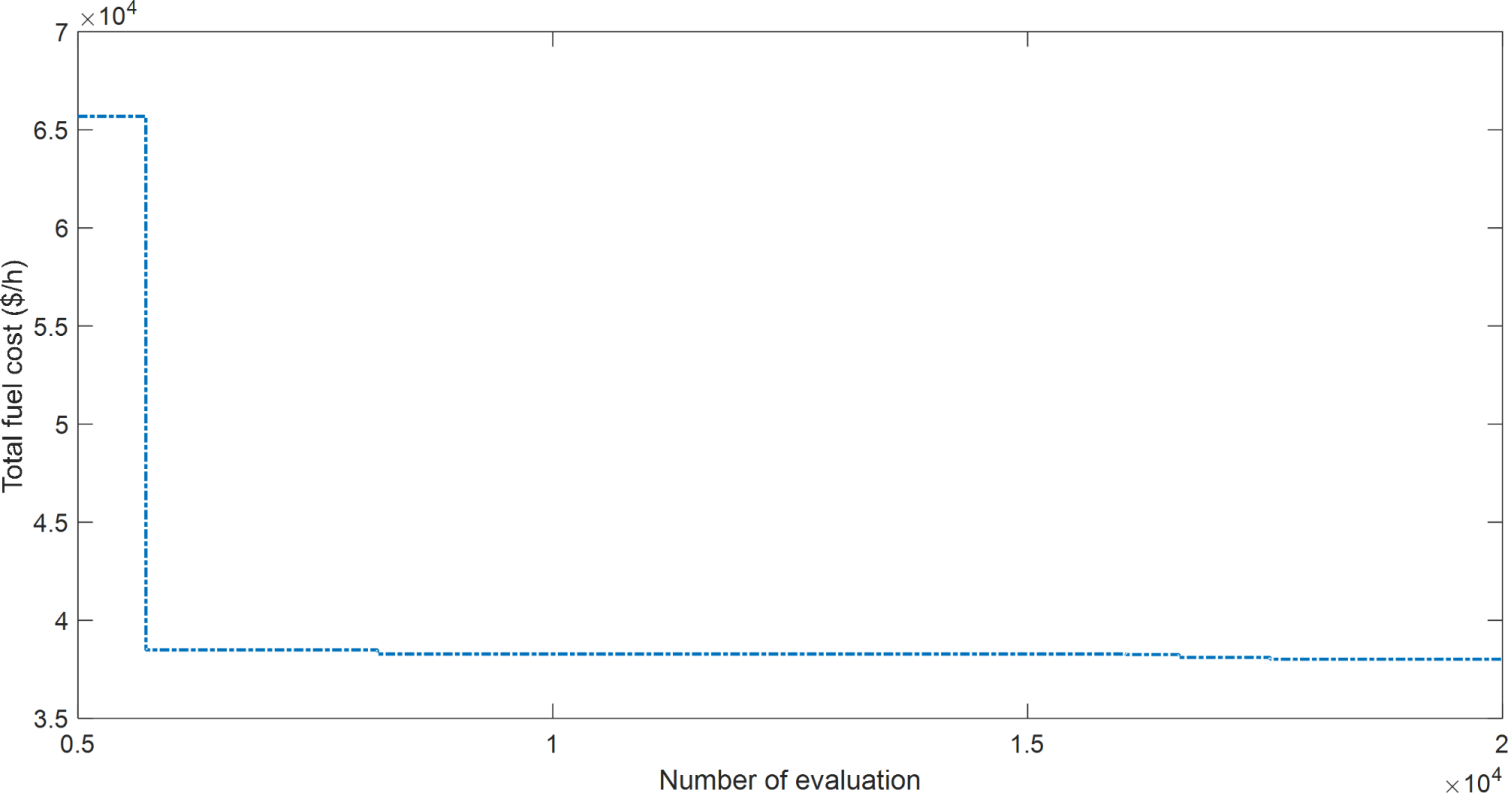
Convergence curve of SAR method based on OPFFF1 for IEEE 57-bus system.

The proposed SAR algorithm is composed of several methods such as practical swarm optimizer (PSO), Moth flam optimizer (MFO), whale and moth flam optimizer (WMFO), grey wolf optimizer (GWO), and cheap optimization algorithm (ChOA). Table 17 displays the best fitness function for OPF based on SAR technique and all comparator methods for OPFFF1, also the power loss is included in this table. Figure 13 explains the convergence curve of SAR method to reach to the best solution of OPFFF1 for IEEE 57-bus system. Based on the recorded data in Table 17; the SAR algorithm achieves the best objective function (total fuel cost) compared with the other methods. The value of fitness function based on SAR technique is equal to 38017.7691758245 $/h. The order of algorithms based on the best fuel cost is SAR, WMFO, MFO, GWO, PSO and ChOA. The order of algorithms based on the power losses is GWO, SAR, ChOA, PSO, MFO, and WMFO. Hence the proposed SAR method has superior performance over all methods applied in this work for the OPFFF1 of IEEE 57-bus system.

**Table 17:** Comparison between SAR method and other methods based on OPFFF1 for IEEE 57-bus system.

Algorithm	Fuel cost	Power losses
SAR	38017.7691758245	21.5490022790274
PSO^58^	42587.218	26.541
GWO^58^	42406.446	20.653
MFO^58^	41397.039	29.513
ChOA^58^	42863.921	25.028
WMFO^58^	39359.123	31.796

#### Results of OPFFF4 for IEEE 57-bus system

The main fitness function discussed in this subsection is minimizing the total cost of fuel. Table 18 displays the best configurations for OPF based on SAR technique for IEEE 57-bus system based on OPFFF4, that include the optimum objective function, the fuel cost, voltage deviations, power loss, and control parameters settings.

**Table 18:** Best parameters solution of OPF problem extracted from SAR method based on OPFFF4 for IEEE 57-bus system.

Parameters	Units	Best value
P1	MW	9.35306308222966
P2	MW	97.2005930412181
P3	MW	4.73594910634648
P6	MW	92.5356343379376
P8	MW	420.214467860132
P9	MW	36.3273731019229
P12	MW	372.226902756100
VG1	$$\:p.u$$	1.01245078599529
VG2	$$\:p.u$$	1.04703820490466
VG3	$$\:p.u$$	1.09162670600981
VG6	$$\:p.u$$	1.07466065173269
VG8	$$\:p.u$$	1.00143357392005
VG9	$$\:p.u$$	0.957153096032730
VG12	$$\:p.u$$	1.02925693045474
T(4–18)	--	0.999097898729473
T(4–18)	--	0.961149358622265
T(21 − 20)	--	0.922860077601741
T(24–25)	--	0.976200460881450
T(24–25)	--	1.07622483749020
T(24–26)	--	1.09618567080478
T(7–29)	--	1.06719937027143
T(34 − 32)	--	1.09010340653652
T(11–41)	--	0.924892358392570
T(15–45)	--	0.928728393319768
T(14–46)	--	0.981092464144505
T(10–51)	--	0.926148269334560
T(13–49)	--	0.900962960154972
T(11–43)	--	0.990201146803200
T(40–56)	--	0.985010866900965
T(39–57)	--	0.979723758645620
T(9–55)	--	1.04733457250927
QC18	MVAR	2.12011988031527
QC25	MVAR	27.3021690341606
QC53	MVAR	28.1225098699415
Multi-objective fitness function		34207.5535252330
Total cost of fuel in ($/h)		33772.3031281152
Power losses in MW		28.6592639736805
Voltage deviation		2.17625198558870

**Fig. 14:**
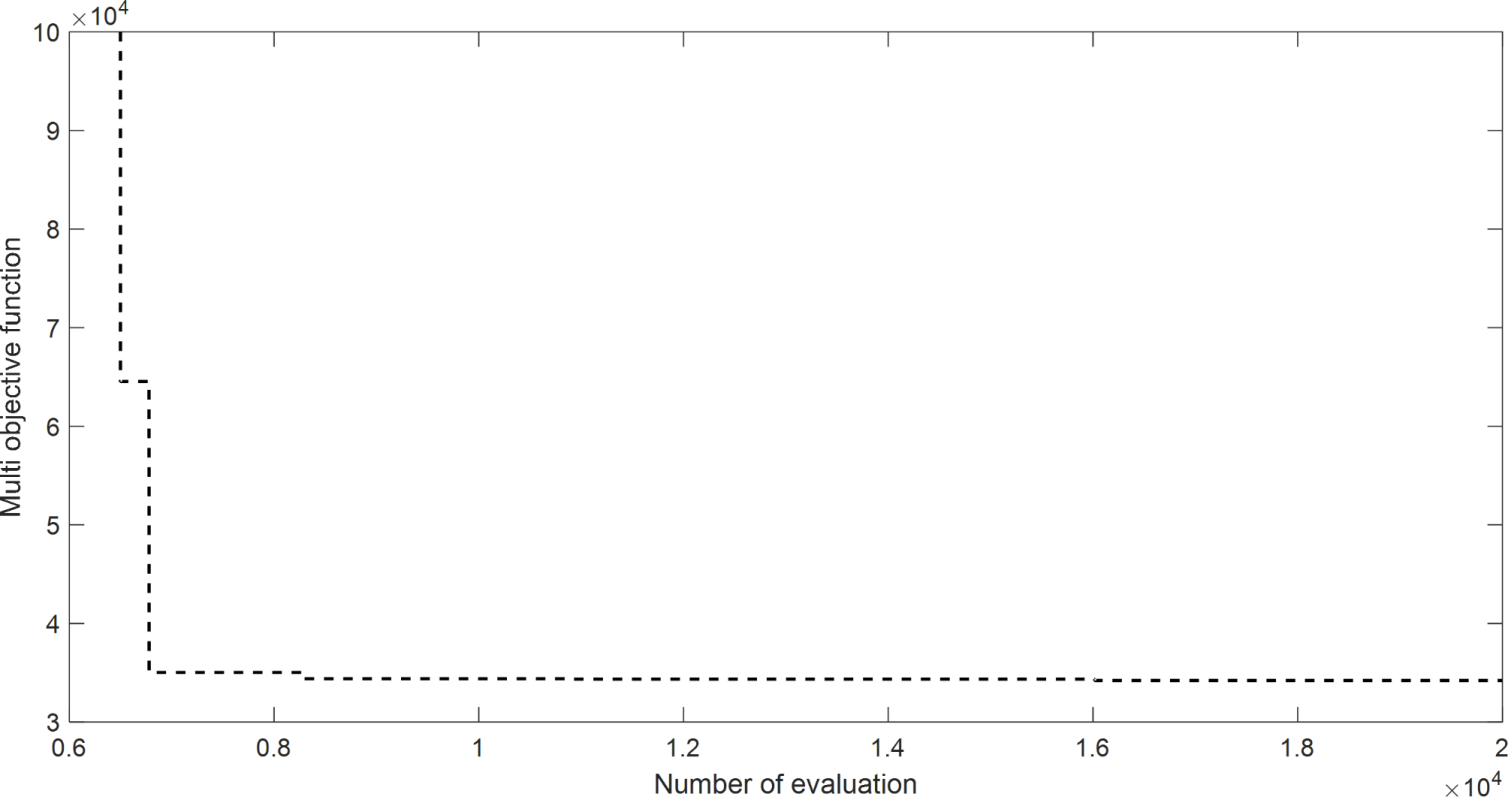
Convergence curve of SAR method based on OPFFF4 for IEEE 57-bus system.

The proposed SAR algorithm is composed of several methods such as practical swarm optimizer (PSO), Moth flam optimizer (MFO), whale and moth flam optimizer (WMFO), grey wolf optimizer (GWO), and cheap optimization algorithm (ChOA). Table 19 displays the best fitness function for OPF based on SAR technique and all comparator methods for OPFFF1, also the power loss is included in this table. Figure 14 explains the convergence curve of SAR method to reach to the best solution of OPFFF4 for IEEE 57-bus system. Based on the recorded data in Table 19; the SAR algorithm achieves the best total fuel cost compared with the other methods. The value of fitness function based on SAR technique is equal to 33772.3031281152 $/h. The order of algorithms based on the best fuel cost is SAR, WMFO, GWO, MFO, PSO and ChOA. The order of algorithms based on the power losses is PSO, ChOA, SAR, MFO, GWO, and WMFO. Hence the proposed SAR method has superior performance over all methods applied in this work for the OPFFF4 of IEEE 57-bus system.

**Table 19:** Comparison between SAR method and other methods based on OPFFF4 for IEEE 57-bus system.

Algorithm	Fuel cost	Voltage deviation	Power losses
SAR	33772.3031281152	2.17625198558870	28.6592639736805
PSO^58^	42465.231	1.833	23.207
GWO^58^	41979.049	1.186	44.435
MFO^58^	42289.258	1.307	32.944
ChOA^58^	42975.547	2.204	24.779
WMFO^58^	41811.734	0.909	51.366

## Conclusion and future work

The OPF problem in electric power systems has been successfully solved by using the suggested SAR, as this article has demonstrated. For a quick, precise, and optimized solution to the OPF problem, the SAR has been validated. The SAR is evaluated and compared to determine the best algorithm to schedule control variables on the IEEE 30-bus, IEEE 14-bus and IEEE 57-bus standard benchmark networks in order to guarantee reduced fuel costs, power losses and an enhanced voltage deviation as a single fitness function for every case. Four objectives are tested in this work: an economic problem (i.e., reducing the total fuel costs associated with power production); an operational issue regarding the voltage deviation; a practical challenge regarding the power losses; and a practical challenge regarding the cost in addition to the voltage deviation as a multi-objective function. The suggested SAR algorithm is compared to other optimization techniques, including the gradient method, artificial bee colony, gravitational search algorithm, modified shuffle frog leaping algorithm, biogeography-based optimization, Jaya algorithm, enhanced genetic algorithm, and differential evolution algorithm, to highlight the effectiveness and potential of the SAR algorithm. The SAR approach is superior as evidenced by the obtained results for the OPF compared to all rival algorithms in every fitness function situation. The value of minimum power losses based on SAR technique is equal to 0.459733441487247 MW and 2.71286428848434 MW for IEEE 14-bus and IEEE 30-bus respectively. The value of minimum fuel cost based on SAR technique is equal to 8051.12225602148 $/h, 798.197578585806 $/h and 38017.7691758245 $/h for IEEE 14-bus, IEEE 30-bus and IEEE 57-bus system respectively. The value of minimum voltage deviation based on SAR technique is equal to 0.0357680148269292 and 0.0978069572088536 for IEEE 14-bus and IEEE 30-bus respectively. The same optimization method can be used for other purposes, such as optimal power flow with multi-objective function, the best location and size for Fixed/Switched Capacitive Banks, and the best placement and size for Distributed Renewable Energy within smart grids that use FACTS devices to minimize feeder losses and guarantee the best possible integration of renewable energy systems.

## Data Availability

The data sets provided during the current study are available when requested from the corresponding author.
